# *Tg(Th-Cre)FI172Gsat* (*Th-Cre*) defines neurons that are required for full hypercapnic and hypoxic reflexes

**DOI:** 10.1242/bio.026823

**Published:** 2017-07-06

**Authors:** Jenny J. Sun, Russell S. Ray

**Affiliations:** Baylor College of Medicine, Department of Neuroscience, 1 Baylor Plaza, T707, Houston, TX 77030, USA

**Keywords:** Brainstem, Catecholamine, DREADD, Hypercapnic reflex, Hypoxic reflex, Respiration

## Abstract

The catecholaminergic (CA) system has been implicated in many facets of breathing control and offers an important target to better comprehend the underlying etiologies of both developmental and adult respiratory pathophysiologies. Here, we used a noninvasive DREADD-based pharmacogenetic approach to acutely perturb *Tg(Th-Cre)FI172Gsat* (*Th-Cre*)-defined neurons in awake and unrestrained mice in an attempt to characterize CA function in breathing. We report that clozapine-N-oxide (CNO)-DREADD-mediated inhibition of *Th-Cre*-defined neurons results in blunted ventilatory responses under respiratory challenge. Under a hypercapnic challenge (5% CO_2_/21% O_2_/74% N_2_), perturbation of *Th-Cre* neurons results in reduced f_R_, 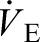 and 
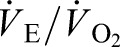
. Under a hypoxic challenge (10% O_2_/90% N_2_), we saw reduced f_R_, 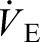 and 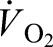, in addition to instability in both interbreath interval and tidal volume, resulting in a Cheyne-Stokes-like respiratory pattern. These findings demonstrate the necessity of *Th-Cre*-defined neurons for the hypercapnic and hypoxic ventilatory responses and breathing stability during hypoxia. However, given the expanded non-CA expression domains of the *Tg(Th-Cre)FI172Gsat* mouse line found in the brainstem, full phenotypic effect cannot be assigned solely to CA neurons. Nonetheless, this work identifies a key respiratory population that may lead to further insights into the circuitry that maintains respiratory stability in the face of homeostatic challenges.

## Abstract (precis)

**Summary:** DREADD-mediated silencing of *Tg(Th-Cre)FI172Gsat*-defined neurons in adult mice results in reduced O_2_ and CO_2_ breathing reflexes and respiratory rhythm destabilization under hypoxic challenge, resembling Cheyne-Stokes respiration.

## INTRODUCTION

The catecholaminergic (CA) system, including the dopaminergic, noradrenergic and adrenergic systems, has been implicated in respiratory homeostasis (Li et al., 2008; Viemari, 2008). A better understanding of the role of the CA system in breathing is of importance for determining the potential mechanisms in developmental disorders with respiratory features, such as Congenital Central Hypoventilation Syndrome (CCHS) (Ramanantsoa and Gallego, 2013), Rett Syndrome (Katz et al., 2009; Weese-Mayer et al., 2006; Julu et al., 2001) and Sudden Infant Death Syndrome (SIDS) (Weese-Mayer et al., 2007); pathophysiologies such as obstructive sleep apnea (Hakim et al., 2012; Zhu et al., 2007), Acute Respiratory Distress Syndrome (ARDS) (Beitler et al., 2016) and Chronic Obstructive Pulmonary Disorder (COPD) (Williams et al., 2014; Yamauchi et al., 2012), where pulmonary dysfunction leads to hypoxia and respiratory destabilization; as well as to inform upon opiate-mediated respiratory arrest (Lalley, 2008). CA neurons are defined by their expression of tyrosine hydroxylase (Th), an early synthesizing enzyme in the CA pathway, leading to production of dopamine, noradrenaline and adrenaline, encompassing several populations in the nervous system. Here, we aimed to characterize the CA system in respiratory function in adult mice through the use of acute and targeted pharmacogenetic neuronal inhibition.

DREADD-mediated neuronal manipulations combined with recombinase based targeting strategies have provided an approach for functional circuit mapping that enables acute noninvasive perturbation of targeted populations while measuring respiratory output in conscious and unrestrained adult mice (Hennessy et al., 2017; Brust et al., 2014; Ray et al., 2012, 2011). The inhibitory pharmacogenetic DREADD or hM4Di receptor is inactive until activated by administration of the biologically inert ligand clozapine-N-oxide (CNO), to perturb neuron firing. To examine the potential aspects of respiratory physiology served by the CA system, we utilized the Cre-responsive *RC::PDi* mouse in combination with the *B6.FVB(Cg)-Tg(Th-Cre)FI172Gsat* (hereafter *Th-Cre*) driver to express the hM4D receptor in CA and other neurons defined by this frequently used *Th-Cre* driver. Using whole-body plethysmography, we assessed respiratory and metabolic function under baseline (21% O_2_/79% N_2_), hypercapnic (5% CO_2_/21% O_2_/74% N_2_) and hypoxic (10% O_2_/90% N_2_) conditions after CNO-DREADD-mediated perturbation of targeted cells, examining several aspects of respiratory and metabolic homeostasis, including rate (f_R_), tidal volume (V_T_), minute ventilation 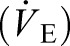, oxygen consumption 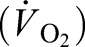 and waveform patterning.

Our results show that hM4Di-mediated inhibition of *Th-Cre*-defined neurons results in reduced hypercapnic and hypoxic reflexes as well as temperature deficits, identifying a population of cells that are critical for maintaining respiratory and metabolic homeostasis. However, because of additional *Th-Cre* expression domains in multiple populations, we cannot fully attribute the respiratory phenotypes seen here solely to the CA system. Nonetheless, the results presented provide an important neural correlate for further study of ventilation, metabolic drive and rhythm stability in the hypoxic and hypercapnic ventilatory responses, toward a better understanding of potential neural mechanisms involved in hypoxic disordered breathing.

## RESULTS

### The *B6.FVB(Cg)-Tg(Th-Cre)FI172Gsat* driver defines cells required for the hypercapnic ventilatory response

**Fig. 1.**
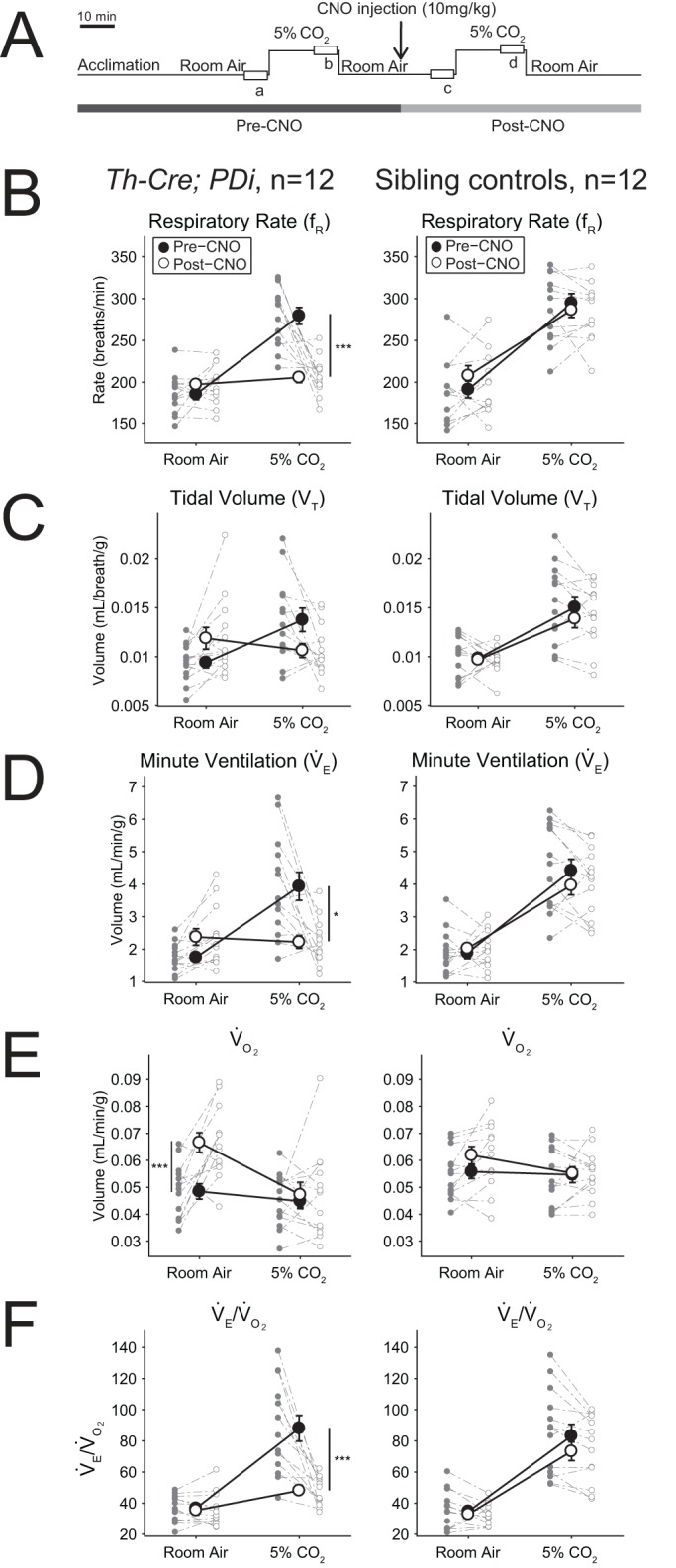
***Th-Cre; PDi* mice show a blunted response to hypercapnic conditions after DREADD-mediated acute perturbation.** (A) Hypercapnic protocol. (B-F) CNO-DREADD-mediated inhibition of *Th-Cre; PDi*-defined neurons results in changes in room air ventilation and decreased hypercapnic ventilation. Upon CNO administration, *Th-Cre; PDi* animals (*n*=12) show an increase in 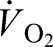 (*P*=0.01) but no overall change to 
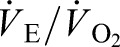
. Under 5% CO_2_ conditions, CNO administration in *Th-Cre; PDi* animals results in dramatically reduced f_R_ (*P*<0.001), 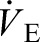 (*P*=0.01) and 
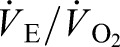
 (*P*<0.001). Sibling controls (*n*=12) show no significant difference between pre- and post-CNO conditions. Individual data points and mean±s.e.m. are shown on each graph. Statistical significance was determined using a linear mixed-effects regression model with animal type (experimental or control) and CNO treatment (pre or post) as fixed effects and animal ID as a random effect. *P<0.05; **P<0.01; ***P<0.001; ns, not significant.

**Fig. 2.**
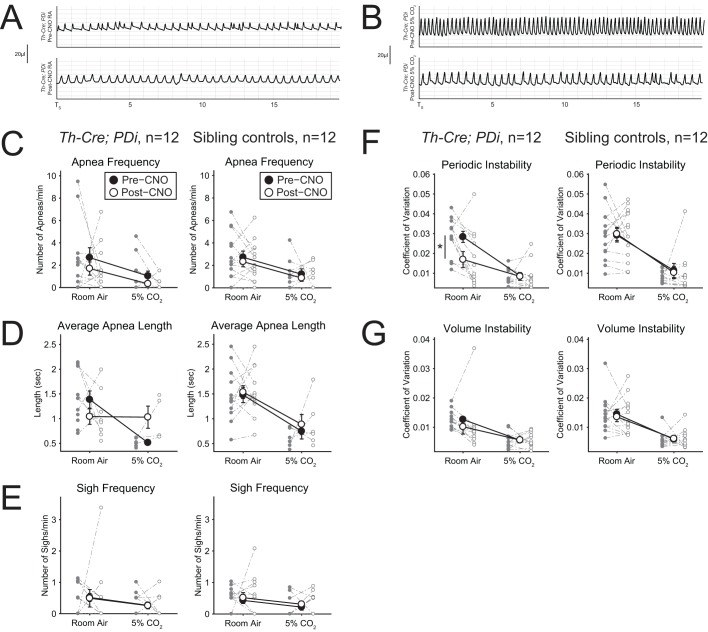
***Th-Cre; PDi* mice have minimal changes in waveform pattern after DREADD-mediated acute perturbation under room air and hypercapnic conditions.** (A) Representative respiratory traces pre- and post-CNO during room air breathing conditions in *Th-Cre; PDi* animals. (B) Representative respiratory traces pre- and post-CNO during hypercapnic breathing conditions in *Th-Cre; PDi* animals. (C-G) *Th-Cre; PDi* animals (*n*=12) showed a reduction in periodic instability (F) after CNO administration under baseline conditions (*P*=0.017) and no change in waveform parameters (apnea frequency, average apnea length, sigh frequency, periodic and volume instability) under hypercapnic conditions. Sibling controls (*n*=12) showed no significant difference before or after CNO administration. Individual data points and mean±s.e.m. are shown on each graph. Statistical significance was determined using a linear mixed-effects regression model with animal type (experimental or control) and CNO treatment (pre or post) as fixed effects and animal ID as a random effect.*P<0.05; **P<0.01; ***P<0.001; ns, not significant.

To assess the requirement of *Th-Cre*-defined neurons for the increased ventilatory response to hypercapnic (high CO_2_) conditions, we employed the established *RC::PDi* inhibitory DREADD system and partnered it with the transgenic *Th-Cre* allele that captures CA neurons, in addition to several other brainstem populations, listed in Table 1. Using whole-body plethysmography, we measured the ventilatory response of animals under room air and 5% CO_2_ before and after CNO administration (Fig. 1A). *Th-Cre; PDi* animals had baseline respiratory parameters similar to sibling controls during room air and 5% CO_2_ conditions prior to CNO administration. In contrast, after CNO administration, *Th-Cre; PDi* mice showed significant respiratory and metabolic changes, especially under hypercapnic conditions (Fig. 2A,B). Under room air conditions, compared to pre-CNO values, we saw a statistically insignificant trend of increased 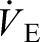 (Fig. 1D, +35.9%, *P*=0.83) mediated by small increases in both f_R_ (Fig. 1B, +6.28%, *P*=0.68) and V_T_ (Fig. 1C, +26.2%, *P*=0.83). However, overall 
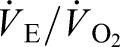
 was unchanged as 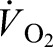 increased (Fig. 1E, +27.9%, *P*=0.01). We also observed a reduction in periodic instability [Fig. 2F, −40.5% reduction in interbreath interval (IBI) coefficient of variation (CV), *P*=0.017]. Upon a post-CNO hypercapnic challenge, 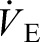 was significantly reduced compared to pre-CNO values (Fig. 1D, −43.6%, *P*=0.01), mediated by a large reduction in f_R,_ (Fig. 1B, −26.3%, *P*<0.001) and a small but statistically insignificant reduction in V_T_ (Fig. 1C, −22.7%, *P*=0.12). Overall 
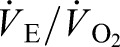
 (Fig. 1F, −69.9%, *P*<0.001) was also significantly reduced as post-CNO 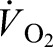 levels were similar to pre-CNO levels. Other than the decrease in periodic instability seen under room air conditions, *Th-Cre; PDi* animals did not show significant variability between pre- and post-CNO respiratory patterns under either room air conditions or the 5% CO_2_ hypercapnic challenge (Fig. 2).

**Table 1.**
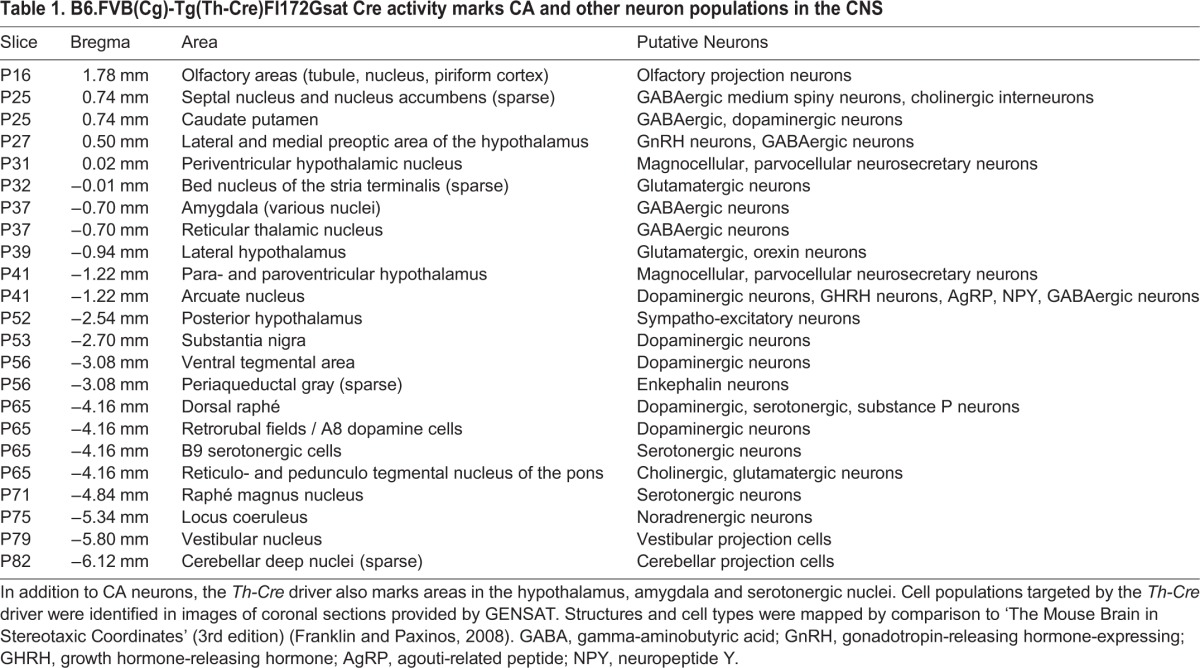
**B6.FVB(Cg)-Tg(Th-Cre)FI172Gsat Cre activity marks CA and other neuron populations in the CNS**

Sibling control *PDi* mice showed no significant difference before or after CNO administration in 
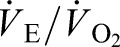
, or constituent f_R_, V_T_, 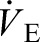, or 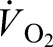 parameters or waveform pattern under room air or hypercapnic challenge.

### The *B6.FVB(Cg)-Tg(Th-Cre)FI172Gsat* driver defines cells required for the hypoxic ventilatory response

**Fig. 3.**
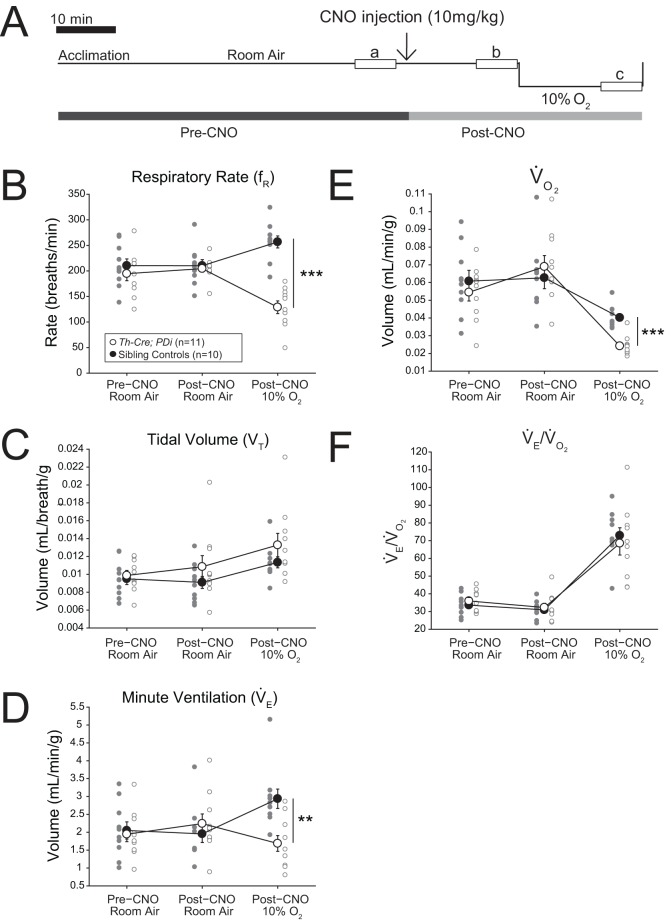
***Th-Cre; PDi* mice show a blunted response to hypoxic conditions after DREADD-mediated acute perturbation.** (A) Hypoxic protocol. (B-F) CNO-DREADD-mediated inhibition of *Th-Cre; PDi*-defined neurons results in decreased hypoxic ventilation. Upon CNO administration, no change is seen under room air conditions in *Th-Cre; PDi* mice (*n*=11). Under hypoxic conditions, *Th-Cre; PDi mice* show dramatically reduced f_R_ (*P*<0.001), 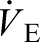 (*P*=0.0013) and 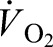 (*P*<0.001) as compared to sibling controls (*n*=10). Sibling controls show no significant difference before or after CNO administration under room air conditions. Individual data points and mean±s.e.m. are shown on each graph. Statistical significance was determined using a linear mixed-effects regression model with animal type as a fixed effect. *P<0.05; **P<0.01; ***P<0.001; ns, not significant.

**Fig. 4.**
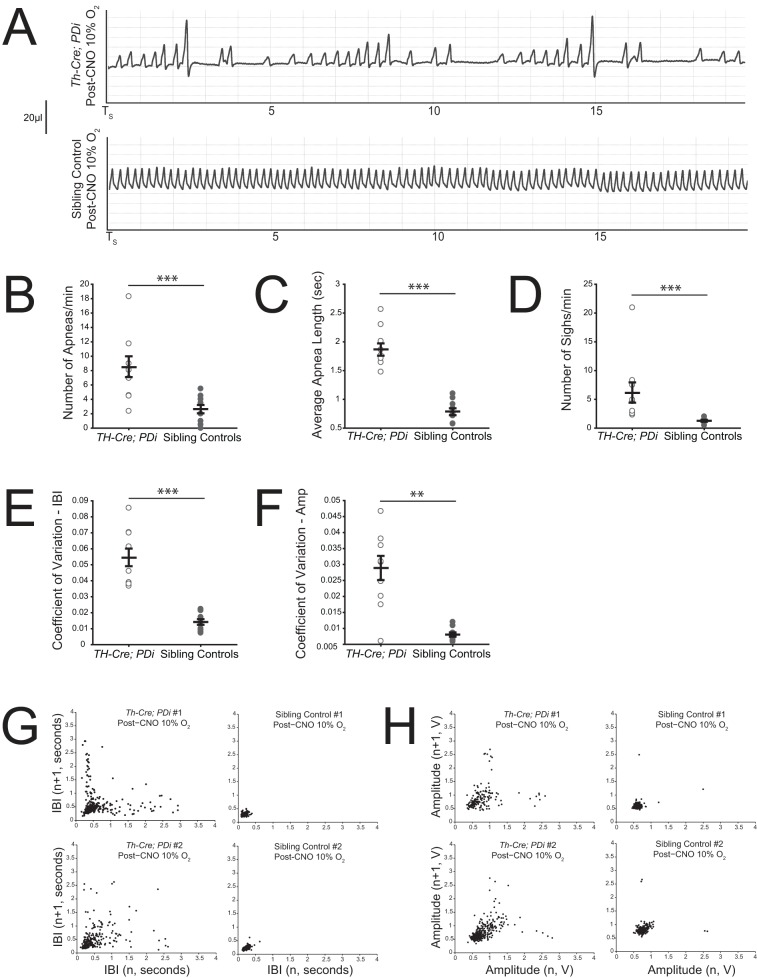
***Th-Cre; PDi* mice show dramatic changes** **in waveform pattern under hypoxic conditions after DREADD-mediated acute perturbation.** (A) Representative respiratory traces post-CNO during hypoxic breathing conditions in *Th-Cre; PDi* animals and sibling controls. In addition to reductions in amplitude and frequency, significant instability in both periodicity and amplitude can be seen in *Th-Cre; PDi* animals. (B-F) *Th-Cre; PDi* animals (*n*=11) showed a dramatic change in waveform pattern after CNO administration under hypoxic conditions, with increases in apnea number (*P*=0.01), average apnea length (*P*<0.001), sigh number (*P*=0.01), and periodic (*P*<0.001) and volume instability (*P*<0.001) as compared to sibling controls (*n*=10). Individual data points and mean±s.e.m. are shown on each graph. Statistical significance was determined using a linear mixed-effects regression model with animal type as a fixed effect. *P<0.05; **P<0.01; ***P<0.001; ns, not significant. (G) Poincaré plots (IBI plotted as a function of the previous IBI) for two *Th-Cre; PDi* mice (left) and two sibling controls (right) after CNO administration while breathing 10% O_2_. (H) Poincaré plots (the amplitude plotted as a function of the previous breath's amplitude) for two *Th-Cre; PDi* mice (left) and two sibling controls (right) after CNO administration while breathing 10% O_2_.

To assess the requirement of *Th-Cre*-defined neurons for the increased ventilatory response to hypoxic (low oxygen) conditions, we also measured respiration of *Th-Cre; PDi* animals under room air and 10% O_2_ conditions (Fig. 3A). Mirroring the data from the hypercapnic challenge, *Th-Cre; PDi* mice showed a statistically insignificant trend of increased 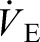 (Fig. 3D, +21.6%, *P*=0.22), mediated by small increases in f_R_ (Fig. 3B, +5.1%, *P*=0.57) and V_T_ (Fig. 3C, +14.0%, *P*=0.15), under room air after CNO administration as compared to pre-CNO values. Overall 
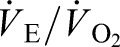
 was unchanged once again as there was a parallel increase in 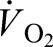 (Fig. 3E, +26.6%, *P*=0.22). Compared to post-CNO sibling controls, *Th-Cre; PDi* mice showed a robustly reduced ventilatory hypoxic response (Fig. 3) with reductions in f_R_ (Fig. 3B, −49.8%, *P*<0.001), 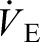 (Fig. 3D, −46.3%, *P*=0.0013) and 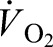 (Fig. 3E, −39.8%, *P*<0.001). The change in 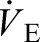 and 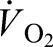 was matched at 15 min as the 
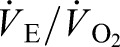
 ratio was not significantly different (Fig. 3F). Poincaré analysis at 15 min of 10% O_2_ reveals significant respiratory instability (Fig. 4A,G,H) in the *Th-Cre; PDi* animals as compared to sibling controls in both interbreath interval (Fig. 4E, *P*<0.001 for CV comparison) and tidal volume (Fig. 4F, *P*<0.001), culminating in a significant increase in apneas (Fig. 4B, *P*=0.01), apnea length (Fig. 4C, *P*<0.001), and sighs (Fig. 4D, *P*=0.01).

Sibling control *PDi* mice showed no significant differences before or after CNO administration in 
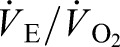
, or constituent f_R_, V_T_, 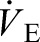, or 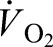 parameters or waveform pattern under room air.

### The *B6.FVB(Cg)-Tg(Th-Cre)FI172Gsat* driver defines cells required for temperature maintenance

**Fig. 5.**
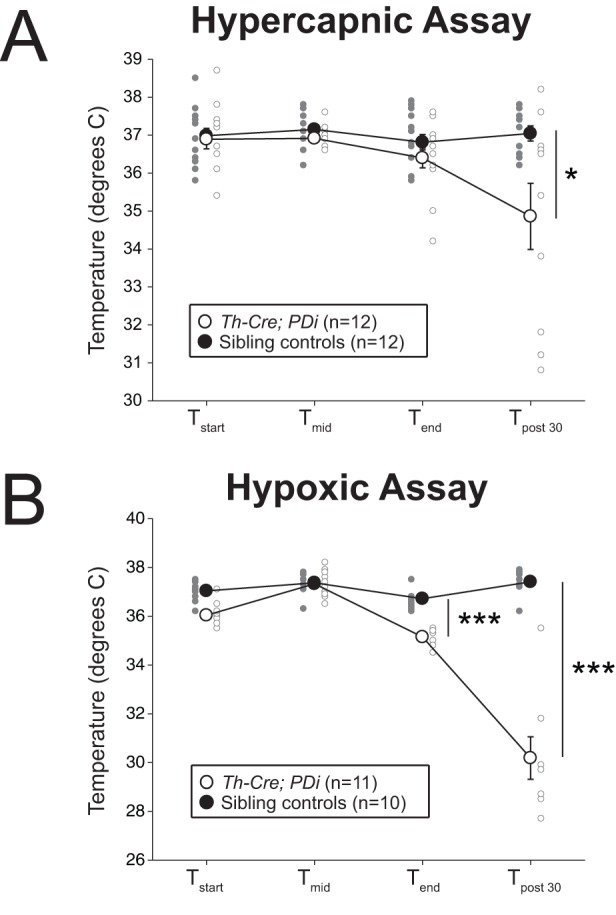
***Th-Cre; PDi* mice show a reduced temperature following CNO administration.** (A) 30 min after the end of the hypercapnic assay (T_post 30_), *Th-Cre; PDi* animals showed a small reduction in temperature as compared to sibling controls (*P*=0.025). (B) After hypoxic exposure, *Th-Cre; PDi* animals showed a reduction in temperature at the end of the assay (T_end_, *P*<0.001) and a dramatic drop 30 min after the end of the assay (T_post 30_, *P*<0.001). Individual data points and mean±s.e.m. are shown on each graph. Statistical significance was determined using a linear mixed-effects regression model with animal type as a fixed effect. *P<0.05; **P<0.01; ***P<0.001; ns, not significant.

Temperature was taken as an element of the respiratory protocol at the beginning of the assay (pre-CNO at ambient temperature, ∼23°C), immediately before CNO injection (pre-CNO at 30°C), immediately after the end of the assay (post-CNO at 30°C), and 30 min after the end of the assay (post-CNO at ambient temperature, ∼23°C). In both the hypercapnic and hypoxic assays, *Th-Cre; PDi* animals showed a significant reduction in body temperature 30 min after the end of the assay as compared to sibling controls (Fig. 5, *P*=0.025 and *P*<0.001**)**. Additionally, they also showed a reduced temperature immediately after hypoxic exposure (*P*<0.001).

## DISCUSSION

The goal of this project was to build a model system that captures CA populations for functional perturbation to identify and study circuits involved in respiratory homeostasis. To access the CA system, we utilized the *RC::PDi* mouse line to express the inhibitory DREADD receptor in *B6.FVB(Cg)-Tg(Th-Cre)FI172Gsat*-defined neurons and cells. Upon intraperitoneal injection of CNO, DREADD activation is expected to activate downstream endogenous Kir channels through G protein signaling and hyperpolarize neurons, disrupting their function and enabling acute whole-body barometric plethysmography studies on conscious and unrestrained animals.

The *Th-Cre* driver mouse line captures populations that are critical to both the hypercapnic and hypoxic ventilatory responses when perturbed by exogenous Gi signaling via CNO-activated DREADD (hM4D or Di). During hypercapnic challenges, CNO-DREADD-mediated perturbation results in reduced f_R_, 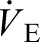 and 
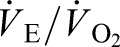
, and during hypoxic challenges, perturbation results in reduced f_R_, 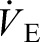, and 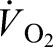. Additionally, while statistically insignificant, in room air conditions of both studies, perturbation of *Th-Cre* cells resulted in a trend of increased 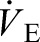 with a matched increase in 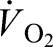 (that was significant in the hypercapnic assay). These results suggest that *Th-Cre* captures a population of cells that may directly or indirectly influence metabolic rate, but does not disrupt, under baseline conditions, 
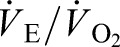
 regulation.

Interestingly, perturbation of *Th-Cre* neurons under hypoxic conditions also results in Cheyne-Stokes-like respiration with instability in both interbreath interval and tidal volume and an increased number of apneas and sighs. This suggests that the *Th-Cre* driver captures neuron populations involved in central breathing pattern generation, the functions of which are most critical under hypoxic stress as the phenotype is not seen in normoxic or hypercapnic conditions. The shape of the waveform suggests that postinspiratory and/or expiratory populations are affected.

To avoid the potential confound of a hypoxic plasticity response affecting post-CNO room air and hypoxic ventilation, we did not include a pre-CNO hypoxic exposure in our protocol. Thus, we cannot technically rule out the possibility that the hypoxic phenotype we observe is not due to CNO-mediated perturbation of the targeted cells, but a background interaction between the genetic background of the *Th-Cre* mouse and hypoxia, or the genetic background and injection or CNO. We find this highly unlikely, however, considering the type and magnitude of the phenotype, which we have never observed in a multitude of our own DREADD-based assays, including other drivers on the GENSAT genetic background, and several previous reports showing CNO to be otherwise inert in these assays.

The *Th-Cre* driver captures several different populations of neurons in the brainstem and elsewhere in the central nervous system (CNS) beyond CA neurons, including subsets of the serotonergic raphé nuclei, the vestibular nucleus, and the reticulo- and pedunculo-tegmental nuclei of the pons (Table 1). Our data using the same hM4D-CNO system in combination with a dopaminergic-specific *Dopamine-active-transporter (DAT)-Cre*, a gene knock in with high fidelity, and a noradrenergic/adrenergic-specific *Dopamine-beta-hydroxylase (DBH)-Cre*, also show deficits in the hypercapnic response but do not phenocopy the hypoxic deficit and instability (data not shown), suggesting that distinct aspects of the phenotype seen in *Th-Cre; PDi* animals may arise from a non-CA population of cells.

Given the large number of anatomically defined populations captured in the CNS as well as possible peripheral populations, it becomes difficult to speculate on which populations may contribute to which aspects of the observed hypercapnic and hypoxic phenotypes. Further confounds arise from differences in approach. While we use a cell autonomous acute pharmacogenetic perturbation, many approaches utilize genetic, pharmacological and electrolytic lesions that may produce cell nonautonomous effects, induce developmental compensatory mechanisms, and capture fibers of passage and other intercalated cell types. Lastly, our respiratory measurements are normalized to oxygen consumption, which can partially account for indirect or secondary respiratory changes that may result from increased or decreased metabolic drive. Nonetheless, it is likely that we are perturbing several neuron populations that are important to different aspects of breathing in developmental disorders, pathophysiologies and neurodegenerative disorders.

Several developmental disorders such as Rett Syndrome (Katz et al., 2009; Weese-Mayer et al., 2006; Viemari et al., 2005), Pit-Hopkins Syndrome (Peippo and Ignatius, 2012) and CCHS (Ramanantsoa and Gallego, 2013) show respiratory instability and reduced chemosensory function. In these disorders, central serotonergic and noradrenergic chemosensory function has been shown to be affected and appear to be captured by this driver (Toward et al., 2013; Viemari et al., 2005). Although this is an adult model, the findings may offer some insight into the potential role of CA in SIDS, both autonomously and nonautonomously, where CA levels have been shown to be lowered in *PACAP−/−* mice that have a higher level of sudden death in the first 2 weeks of life and show reduced catecholamine levels and chemosensory deficits (Arata et al., 2013; Cummings et al., 2004). Additional pathophysiologies such as obstructive sleep apnea, ARDS and COPD present with respiratory instability while engaging the hypoxic reflex circuitry to maintain vital functions. A series of studies on Parkinson's disease using 6-hydroxydopamine (6-OHDA) in the striatum showed a loss of CA dopamine neurons and retrotrapezoid nucleus (RTN) neurons resulting in chemosensory dysfunction that was mitigated by potential compensation by the locus coeruleus (Tuppy et al., 2015; Oliveira et al., 2017). Given that this model only shows respiratory instability under hypoxic challenges, the *B6.FVB(Cg)-Tg(Th-Cre)FI172Gsat* driver defines new populations that either as a whole or separately may play a role in the circuitry that is developmentally perturbed in these pathophysiologies, or engaged when responding to hypoxic stress from obstruction, compromised pulmonary function or neurodegeneration.

Finally, perturbation of *Th-Cre; PDi*-defined neurons resulted in significant temperature deficits 30 min after removal from the chamber (∼70 min after CNO injection) and a deficit immediately after hypoxic exposure (∼40 min after CNO injection). As thermogenesis is a crucial component of physiological homeostasis, this suggests that the *Th-Cre* driver captures cells critical for temperature maintenance and may play a role in metabolic homeostasis.

## Conclusions

The results presented here define a set of neurons that encompass the CA system and other distinct populations in the brainstem that play a role in room air respiration and metabolic regulation, and are also required for the hypercapnic and hypoxic ventilatory reflexes, and respiratory stability while facing a homeostatic challenge. This work sets the stage for future studies to more closely model respiratory pathophysiologies to determine the critical subsets of neurons required for respiratory stability under hypoxic conditions, yielding key insights as to the neural substrates that underlie congenital and adult respiratory pathophysiologies.

## MATERIALS AND METHODS

### Mice

Studies were approved by the Baylor College of Medicine Institutional Animal Care and Use Committee (IACUC) under protocol AN-6171, and all experiments were performed in accordance with relevant guidelines and regulations.

Heterozygous *B6.FVB(Cg)-Tg(Th-Cre)FI172Gsat* (*Th-Cre*) mice were mated to homozygous *RC::PDi* mice to derive sibling cohorts in which all mice carried the *RC::PDi* allele. *RC::PDi* mice were maintained in the colony by backcrossing to C57BL/6J mice before homozygosing. Sibling mice that did not inherit the *Th-Cre* allele were used as sibling controls to the *Th-Cre; PDi* offspring. All experimental animals were treated in compliance with the United States Department of Health and Human Services and the Baylor College of Medicine IACUC guidelines.

### Whole-body plethysmography

Mouse respiration was measured in a custom built barometric whole-body plethysmograph. Ventilation was calibrated to a series of 20 µl pipetman injections into an empty 140 ml water-jacketed temperature-controlled chamber at a rate of 3 Hz, while measuring baseline gas composition for each assay**.** The rate of gas inflow and outflow was continuously controlled via dual rotameters. Flow rate was 198.11 ml/min. Gas was humidified to 100% by passing through a water column prior to entering the chamber and dehumidified by passing through 20 cm Nafion tubing encased in silica desiccant. Plethymosgraphy pressure changes were measured using a DP45 differential pressure transducer and CD15 carrier demodulator (Validyne, Northridge, USA) in comparison to a reference chamber and recorded with LabChartPro (ADInstruments, Colorado Springs, USA) in real time. Chamber temperature was constantly monitored using a MicroThermo 2 and probe (ThermoWorks, American Fork, USA), and was recorded with LabChartPro in real time. Subsequent waveforms were analyzed offline to determine respiratory rate (f_R_), tidal volume (V_T_), minute ventilation 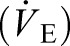, oxygen consumption 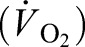 and minute ventilation normalized to oxygen consumption 
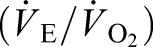
.

### Experimental design

#### Hypercapnic assay

Twelve *Th-Cre; PDi* mice and 12 sibling control mice were assayed for their respiratory response in room air (21% O_2_/79% N_2_) and in 5% CO_2_ balanced with room air before and after CNO administration (Fig. 1A). Ten male and two female *Th-Cre; PDi* mice, and seven male and five female sibling controls, from nine litters were assayed. The animals ranged in age from 3 months to 6 months, and body weight ranged from 19.7 g to 45.2 g. For each animal, the weight and rectal temperature was taken before the animal was placed in the plethysmography chamber (held at ∼31-32°C). After acclimation (typically 20-40 min, indicated by a steady respiratory trace free from movement artifact), a 20-min baseline room air trace was taken, followed by a 20-min exposure to 5% CO_2_, followed by another 20 min of room air to return respiratory parameters to baseline. The animal was then taken out of the chamber for another rectal temperature measurement and intraperitoneal injection of CNO dissolved in saline at 1 mg/ml for an effective concentration of 10 mg/kg. Prior studies suggest that behavioral changes from CNO in transgenic mice expressing DREADDs can take effect within 5-15 min of injection (Alexander et al., 2009; Ray et al., 2011), and clears from plasma within 2 h (Guettier et al., 2009), although phenotypes can be observed for up to 10-15 h. The animal was returned to the chamber for another 20 min of post-CNO room air, 20 min of 5% CO_2_, and a final 20 min of room air before an end temperature was taken. Temperature was taken again 30 min after the end of the assay after the mouse was singly housed at ambient temperature (∼23°C).

#### Hypoxic assay

Eleven *Th-Cre; PDi* mice and 10 sibling control mice were assayed for their respiratory response in room air both before and after CNO administration, and in post-CNO 10% O_2_. Nine male and two female *Th-Cre; PDi* mice, and six male and four female sibling controls, from eight litters were assayed. The animals ranged in age from 4.5 months to 7 months, and body weight ranged from 24.7 g to 50.1 g. The protocol was similar to the hypercapnic assay, but instead of a 5% CO_2_ exposure, the animal was exposed to 10% O_2_ balanced with N_2_ 20 min after CNO administration (Fig. 3A). Animals were not exposed to a pre-CNO 10% O_2_ condition to avoid potential confounds of hypoxic plasticity.

### Data analysis

Respiratory waveforms were collected when the mouse was at rest, and readings were free from movement artifacts. A minimum of 1 min cumulative data compiled from traces at least 5 s long from the last 5 min of a given experimental condition were analyzed. No filtering or smoothing was applied to the pressure waveform. Tidal volume (V_T_) was determined as described (Ray et al., 2011). Oxygen consumption was determined by comparing the gas composition between calibration in an empty chamber and live breathing using an AEI oxygen sensor and analyzer (AEI Technologies, Pittsburgh, USA).

Poincaré plots and apnea and sigh measurements were determined using at least 1 min of movement-free traces from each breathing condition. Apneas were defined as an IBI that was more than twice as long as the average IBI. Sighs were defined as a breath that had more than twice the amplitude of the average breath. The CV of the IBI and amplitude were also calculated from the same trace compilation of each breathing condition (standard error IBI or amplitude/mean IBI or amplitude) and defined as the periodic and volume instabilities, respectively.

### Statistics

Room air and hypercapnic results were compared using a linear mixed-effects regression model with animal type (experimental or control) and CNO treatment (pre or post) as fixed effects and animal ID as a random effect. Hypoxic and temperature results were compared between animal types using a linear mixed-effects regression model with animal type as a fixed effect. Individual data points and mean±s.e.m. are shown on each graph in all figures.

### CNS Cre activity pattern

Cell populations targeted by the *Tg(Th-Cre)FI172Gsat* driver were identified in images of coronal sections provided by the Gene Expression Nervous System Atlas (GENSAT). Structures and cell types were mapped by comparison to Franklin and Paxinos (2008).
